# Building global capacity for brain and nervous system disorders research

**DOI:** 10.1038/nature16037

**Published:** 2015-11-19

**Authors:** Linda B. Cottler, Joseph Zunt, Bahr Weiss, Ayeesha Kamran Kamal, Krishna Vaddiparti

**Affiliations:** 1 Department of Epidemiology, College of Public Health and Health Professions and College of Medicine, University of Florida, Gainesville, Florida, USA; 2 Department of Neurology, University of Washington, Seattle, Washington, USA; 3 Department of Psychology and Human Development, Vanderbilt University, Nashville, Tennnesse, USA; 4 Stroke Service, Section of Neurology, Department of Medicine, The International Cerebrovascular Translational Clinical Research Training Program (Fogarty International Center, NIH) Aga Khan University, Karachi, Pakistan

## Abstract

The global burden of neurological, neuropsychiatric, substance-use and neurodevelopmental disorders in low- and middle-income countries is worsened, not only by the lack of targeted research funding, but also by the lack of relevant in-country research capacity. Such capacity, from the individual to the national level, is necessary to address the problems within a local context. As for many health issues in these countries, the ability to address this burden requires development of research infrastructure and a trained cadre of clinicians and scientists who can ask the right questions, and conduct, manage, apply and disseminate research for practice and policy. This Review describes some of the evolving issues, knowledge and programmes focused on building research capacity in low- and middle-income countries in general and for brain and nervous system disorders in particular.

Despite the current global burden of neurological, mental health (neuropsychiatric), developmental and substance use (NMDS) disorders, which is projected to increase, there is a lack of well-trained clinicians and scientists who focus on brain and nervous system disorders research in low- and middle-income countries (LMICs)^1–6^ **(**see page S151**)**. This workforce deficiency limits advances in research that can lead to new and improved interventions for those who are living with brain and nervous-system disorders. Notably, there are 200 times more neurologists per capita, and up to 160 times more psychiatrists per capita, in high-income countries than there are in LMICs^7,8^. Brain disorders involve central nervous system (CNS) functioning, making things even more challenging. Key symptoms may involve both internalizing and externalizing behaviour. Internalizing behaviour may be stigmatized and externalizing behaviour may be negatively stereotyped; both may be difficult to treat and associated with poor prognosis. The stigma associated with NMDS disorders applies to both the patients and the clinician researchers who treat them, creating an additional barrier to building and sustaining research capacity.

Thus, for health research in LMICs there is an urgent need to build new, and strengthen existing, individual, institutional and country-wide research capabilities. This Review identifies certain key characteristics of capacity building, and the challenges and lessons learned based on the literature^9,10^ and our own experience.

## BUILDING AND STRENGTHENING RESEARCH CAPACITY

Research capacity building is a systematic, purposeful and goal-oriented effort to strengthen human resources and infrastructure to enable local scientists and institutions to become independent and responsive to existing and emerging health needs and threats^9–13^ . To be sustainable and effective (and to address research-training sustainability concerns) a framework must be created so that research capacity is strengthened and woven together at the individual, institutional and national levels. To create research opportunity and a career pipeline, there needs to be a simultaneous focus on frameworks, goals and opportunities. Embedding research into health systems requires a process that involves competent scientists and a supportive environment that enables research communities to flourish as they use new research tools that contribute to improving the health of the population^12^. This iterative process allows research to be responsive to population needs, and policies and practices to be responsive to research findings.

## INDIVIDUAL LEVEL

At the core of capacity building is the training and mentoring of individuals to design and conduct research; to create or adapt research tools that are relevant to brain-disorders research; to form collaborations with scientists in their institution, elsewhere in the country, and internationally; and eventually to serve as mentors themselves for the next generation of scientists (most effectively if they are within their home countries or region). Increasingly, researchers also need training on how to interact effectively with policy and programme implementers to ensure that they and their practices are adapted to the practices and policies locally.

Capacity building at the individual level begins with mentors who counsel, provide career guidance, and advise on the teaching and sharing of ethical principles that instil integrity in research and care. Research-mentoring strategies and systems are context-driven, and often use apprenticeship or hands-on models, whereby individuals learn by doing. When little in-country research expertise and capacity exists, training may need to take place initially in a higher resource country. However, the goal should always be to have the research training in the context of the trainees’ home institution.

BOX 1 **BIOMEDICAL AND BIOBEHAVIOURAL RESEARCH ADMINISTRATOR DEVELOPMENT PROGRAMME**

The National Institutes of Health (NIH)/National Institute of Child Health and Human Development (NICHD) in collaboration with other NIH Institutions and Centers has made available the Biomedical/Biobehavioral Research Administrator Development (BRAD; http://www.nichd.nih.gov/about/org/od/ohe/brad/Pages/overview.aspx) programme to establish new or strengthen existing offices of sponsored programmes (OSPs) or similar entities in low- and middle-income countries (LMICs). Enhanced OSPs in non-research intensive institutions are essential for the development of enabling and supportive environments in which faculty can develop robust research programmes and provide research experiences for students.

**BRAD objectives**

From a programme perspective, comprehensive and effective research administration is the bridge between research projects and a sustainable research enterprise at LMIC institutions. Accordingly, the BRAD programme objectives are to:

- Encourage and support continuous professional development of OSPs, research administrators and grants managers at all levels.
- Increase the effectiveness and productivity of OSPs (or similar entities) by promoting the use of best practices in research administration.
- Promote OSP sustainability by identifying and addressing barriers to research and by supporting targeted faculty professional development that focuses on increasing competitiveness in obtaining external research support.

In terms of systems, scientists initially educated in LMICs then trained in high-income countries say that they are accustomed to certain ways of providing and receiving feedback, and interacting within hierarchical relationships. When they return to their home academic environments, ideally they will adopt a hybrid mentoring style, combining positive attributes from both the country where they were trained and their home country, of which they have an awareness of local nuances, regarding local customs, politics and bureaucracy.

Questioning and vigorous debate are integral to the scientific process, but young investigators from cultures that emphasize deference in hierarchical relationships may experience a conflict between these two values when they return home.

In terms of research and clinical practice, performing a lumbar puncture to obtain cerebrospinal fluid (CSF), a requirement for the diagnosis of most CNS infections and some degenerative or developmental abnormalities, brain banking and autopsy can all be met with reluctance in LMICs. Extraction of fluids, tissue or organs for donation or banking for research purposes is associated with significant sociocultural barriers in some cultures. Several factors related to religion and culture, and issues related to distrust of the medical system, misunderstandings about religious stances and ignorance often complicate the process. Requests for brain or other organ banking for research purposes could raise concerns that agreeing to donation would discourage doctors from treatment to save lives among relatives or that consenting to banking would result in premature removal of their or their relative’s organs.

To build capacity for these endeavours, mentors must be willing to not just advocate for these techniques, but also to address cultural barriers that may affect policies, as well as the discomfort of trainees who may not have experience of approaching relatives and patients about these procedures.

The National Institutes of Health/Fogarty International Center Global Brain, NIDA International Fellowships and other programmes (see Supplementary Table) are designed to help catalyse research capacity development at an individual level (both as research training opportunities for young investigators, and as research pipeline opportunities for more advanced LMIC investigators) to help prevent the loss of crucial talent and expertise.

## INSTITUTIONAL LEVEL

Institutional capacity building is the administrative foundation and is essential for establishing and sustaining initiatives intended to realize its vision^14^. Research infrastructure includes job positions that provide protected time for research, as well as robust laboratories and clinical spaces where diagnosis, treatment and research can be conducted. Research into brain disorders, especially stroke, CNS infections, trauma and neurodegenerative conditions requires the technology to assess structural neurological abnormalities; for example, computerized tomography or magnetic resonance imaging may be non-existent or prohibitively expensive in many LMIC settings. Although research-training grants typically provide the funding necessary to train new scientists and the equipment to increase laboratory capacity, larger infrastructure capacity-building endeavours, such as acquiring high-cost diagnostic neuroimaging or laboratory equipment, or constructing new laboratories, clinics or classrooms, require the financial commitment of institutions with support from funders (ideally, and eventually, at the country level for maximum sustainability).

Research and grants administration are crucial to the sustainability of research programmes within any institution, but good administrators can enhance the development of research capacity in resource-challenged institutions (Box 1).

### Networks

Research and research-training networks enrich the research environment and build capacity by increasing collaborations and partnerships; expanding institutional perspectives from local and national levels to regional and perhaps the global level; and facilitating ideas exchange, dialogue and universal or standardized protocols for brain research^15^. Such networks are most effective when they attract not only individual scientists and academic institutions, but also non-governmental organizations (NGOs), corporations, policymakers, and/or philanthropists, to sustain and embed the research enterprise within a country that is focused on a health issue, such as NMDS disorders. One example is the neuroscience promotion association APRONES. This association was established by a group of neurologists to share knowledge of diseases of the nervous system in LMICs. Members are from Africa, Europe and the United States. The association encourages collaborative studies around the world, while building networks and ultimately research capacity^16^.

## NATIONAL LEVEL

True sustainability of research capacity and its application requires a national commitment to the research enterprise and implementation of research results at the policy level, as well as a continuing dialogue between health practitioners, policymakers and researchers. However, often it is not until research and research-training networks are established that local government and NGOs recognize the benefit of talent and training to system-wide improvements and national human-resource development^12^. At that point, they take actions to sustain it.

According to the WHO^9^, work in support of the ethical review and public accountability of research is not keeping pace with best practices. Opportunities to create a shared framework for storing and sharing research data, tools and materials, have not been met with the same energy in the area of health as they have in other scientific fields. Furthermore, policymakers rarely understand research priorities or use evidence to inform their decisions.

Without country-level planning and action, along with guidance documents, health research in LMICs may be influenced more by the demands of foreign funders’ research and infrastructure interests than by the health priorities of the host country^17^. In LMICs in general, and Africa specifically, increasing the value of health research requires evidence-informed actions to be taken by relevant authorities to ensure that health research is conspicuous in development agendas. It also requires defining, financing and monitoring a clear national plan for a future research enterprise focused on health. To achieve these goals, policymakers and public health and research-funding institutions can use principles adapted from the WHO Strategy on Research for Health^9^ as a guide. These provide the overall framework for research capacity and include reinforcing the research culture and organization; focusing research on key health challenges by setting priorities; strengthening national health research systems and building capacity; encouraging good research practice (setting standards) and consolidating links between health research and action (translation and evidence-based implementation).

BOX 2 **ANATOMY OF A GLOBAL BRAIN RESEARCH FUNDING PROGRAMME**

Achievements of the National Institutes of Health/Fogarty International Centre coordinated global brain research programme (http://www.fic.nih.gov/About/Staff/Policy-Planning-Evaluation/Pages/fogarty-program-evaluation-brain-disorders.aspx).

The programme supports collaborative empirical research and capacity building on brain and nervous-system diseases and disorders identified by the applicants as relevant public health challenges in their low- and middle-income countries (LMICs).

**Programme’s achievements**

Research conducted over 10 years in 45 LMICs, most of which are in sub-Saharan Africa, Latin America and the Caribbean.

Topics were across the spectrum, from mental health and substance use, to peripheral nervous system trauma and gene–environment interactions.

During the first 10 years of the programme, participants published 435 peer-reviewed articles in 249 unique journals, as well as 14 books or chapters.

Grantees also produced unique tools for clinical assessment in the LMIC context, developed and evaluated new interventions, and identified novel laboratory tools or methods.

Almost half of the projects supported training for people, who were not primary collaborators, in LMICs. The programme supported in-depth instruction for at least 138 scientists, for an average of 23 months.

Projects included training or mentoring at the LMIC (or sometimes a high-income country) site, in skills, methods or procedures that are essential to research, including workshops on specific topics, or clinical or research skills.

Achieved mandatory training in research ethics, which built and sustained capacity in research ethics at most sites.

Needs and opportunities for building and strengthening capacity for brain-disorders research are shown in Table 1. Although not exhaustive, they outline specific valuable approaches that can be used by high-income country and LMIC collaborators.

The Supplementary Table includes some organizations that are investing in research-capacity building for brain disorders. Some long-term examples of programmes specifically focused on building a pipeline that stretches from individual to institutional to national research capacity levels for nervous system diseases and disorders in LMICs are shown in Box 2. These programmes include the US NIH/Fogarty coordinated Global Brain and Nervous System Diseases and

BOX 3 **SEVEN PRINCIPLES FOR STRENGTHENING RESEARCH CAPACITY**

Based on the World Health Organization–TDR ESSENCE good practice document series^10^

1. Network, collaborate, communicate and share experiences
2. Understand the local context and accurately evaluate existing research capacity
3. Ensure local ownership and secure active support
4. Build in monitoring, evaluation and learning from the start
5. Establish robust research governance and support structure, and promote effective leadership
6. Embed strong support, supervision and mentorship structures
7. Think long-term, be flexible and plan for continuity

Disorders Across the Lifespan Research Program and several Fogarty centre sponsored institutional research training programmes (focusing on masters, PhD and postdoctoral level training for LMIC investigators; Supplementary Table).

## ADDRESSING CHALLENGES TO CAPACITY BUILDING

The framework to build the individual, institutional and national capacity described requires principles of engagement, much like those for community engagement^18^. Those most relevant are the seven principles from the ESSENCE good practice document series^10^ (Box 3). These core principles serve as a useful guide for funding agencies, the scientific community and academic institutions on how to move forward as they identify priorities, develop goals and objectives, design programmes and establish partnerships. They are also useful principles to address various challenges in collaborations within and between countries and cultures, such as human, infrastructure, technological and ethical challenges.

### Human capacity challenges

Capacity building across countries and cultures for brain-disorders research is inherently a ‘messy’ processes when we consider the scope of research with global partners across completely different time zones, infrastructures, cultural norms, expectations and organizational research capacities. Differences in language and expression of research ideas can lead to confusion and misunderstanding. When choosing terminology for assessments on depression, for example, well-developed Western assessments use the words ‘feeling blue’ to indicate feelings of sadness. To discuss mania, the term ‘high’ might be used. These terms are idiomatic and do not translate well in many languages. Another example is the need for translators and interpreters in different sites within countries where multiple languages and dialects are spoken.

Research training includes emphasizing flexibility to address such cultural challenges to research and working within local and regional norms (the ESSENCE principles shown in Box 3 can help). For example, when conducting a study on the prevalence of opiate use in Afghanistan, two female interviewers were needed to conduct interviews with the female head of the household. Cultural norms dictated that the female interviewers had to be accompanied by a male team member who would make the first contact with the household^19^. Designing research protocols that account for local cultural norms, while educating the high-income country and LMIC institutional review boards is necessary and builds trust and understanding between collaborators over time. Individual challenges can be resolved through open communication and sharing of expectations at the outset.

### Infrastructure capacity challenges

To conduct research, LMIC institutions require institutional review board committees, grant-management personnel, and data and document management capacities. These capacities vary widely across and within countries, but sufficient capacity in these areas is crucial to ensure fidelity to research protocols. Financial resource limitations and limited access to scientific and technical information are also key challenges.

BOX 4 **BIOETHICS TRAINING RESOURCES**

Resources for international bioethics research training curricula can be found through the Fogarty International Centre (Fogarty) International Research Ethics Education and Curriculum Development Award (or bioethics) programme (http://www.fic.nih. gov/ResearchTopics/Pages/Bioethics.aspx).

Scientist Nandini Kumar was in the first class of trainees funded by the Fogarty programme at the University of Toronto (http://www.fic.nih.gov/News/Examples/Pages/bioethics-india.aspx). After completing the course in 2002, Kumar was successful in her bid for a planning grant, and received a full Fogarty award to implement her programme in 2005. Over the first 7 years, Kumar’s programme trained more than 2,000 scientists and health-care workers. More than 50 of them completed the intensive course and 2 earned master’s degrees. The programme has hosted nearly 34 intensive workshops in 16 Indian cities. Its distance-learning programmes train up to 50 people each year. Many of the graduates have published papers, prepared curriculum for bioethics instruction at their own institutions, presented papers at national and international conferences, served as evaluators and set up or become members of ethics committees. As the field has developed, Kumar has become not only a national leader in bioethics, but also a member of international panels, including the US Presidential Commission for the Study of Bioethical Issues.

To overcome these barriers, high-income country and LMIC partners have strengthened institutional support by setting up meetings with presidents, deans and directors of institutes to advocate for more resources and to become less reliant on outside high-income country funding. Researchers have also set up channels of communication and collaboration whereby they can help each other in the grant application process for Western-based grants.

Investigators have also succeeded in seeking permanent access to library resources through high-income country institutions for their trainees. However, more sustainable access within and across LMICs to bridge the global information divide is needed. One source is HINARI^20^.

### Technology capacity challenges

Information and communication technology (ICT) has become increasingly integrated into research and clinical training. ICT involves a variety of technologies, including low-cost two-way voice, picture and video communication; development of geographic information systems, which are useful for planning interventions and mapping the prevalence of neurological conditions and risk factors^21,22^; Internet- and mobile-phone-based health-related interventions^23^, and Internet- and mobile-phone-based data collection^24–32^.

Online courses and degree programmes that have become incorporated into most high-income country academic institutions have particular utility in LMICs where training infrastructure may be lacking or geographical barriers limit participation in conventionally structured research training programmes. The development of massive open online courses (MOOCs) and bidirectional interactive virtual spaces permit multidisciplinary partnerships between students, faculty and mentors across institutions and countries. These provide new practical opportunities for bidirectional training, presentations and classroom-based discussions around the world (either as live or recorded sessions).

The Internet allows research, clinical training and supervision to take place across the globe, and although the content and quality of the online material is important, the effectiveness of the supervision depends on the quality of the input, and learning ultimately rests on the ability and motivation of the trainee. Internet interventions have the potential to reduce manpower requirements, but without sufficient support, completion rates remain unacceptably low. There is a need to rigorously evaluate the use of these technologies in brain-disorders research training to ensure they are effective, acceptable and culturally relevant.

### Ethical challenges

The field of neuroethics is a component of bioethics that deals with the investigation, treatment and research procedures that involve the human brain and brain science. The International Neuroethics Society (http://www.neuroethicssociety.org) was started to promote research that would benefit people around the world.

Although all research training should include human subject research ethics, teams that focus on brain-disorders research face unique ethical challenges. People with neurological disorders are vulnerable, sometimes cognitively or physically challenged and often stigmatized, which creates special challenges when designing protocols that ensure ethical informed consent. It is essential to address these special challenges (some of which are similar to those faced by researchers working with populations affected by HIV/AIDS) in training curricula for research that is specific to brain disorders. The Fogarty funded Pakistani stroke research training programme has a dedicated neuroethics training module, in which every mentored project that involves mental health research has a bioethics programme (Box 4).

Use of functional magnetic resonance imaging (fMRI), near-infrared recording systems (NIRS), polygraphy to extract information, genetic testing and cognitive enhancement using drugs and brain stimulation are just a few examples of current and evolving technologies that raise moral and ethical questions. The application of these modern techniques has been gaining momentum in the developing world, thereby indicating an urgent need to integrate neuroethics training into the neuroresearch capacity building efforts in LMICs.

### Metrics

A robust set of metrics is crucial to demonstrate the value of research capacity building to diverse stakeholders and to understand how to make research-training activities the most effective. Output measures include educational materials such as courses, modules and workshops; creation and transfer of new knowledge, such as prototypes and innovative protocols; and measuring trainee engagement indicators (for example, number of short-, medium- and long-term trainees taught, number of trainees completing courses or acquiring new skills and trainee feedback). As research training programmes mature, conventional metrics are needed, such as publications, grants, awards, memberships in societies, degrees awarded and faculty appointments.

Measuring the long-term impact of building research capacity is a significant challenge. Many funding agencies have strict guidelines for tracking career successes of funded scholars for up to 15 years after training and evaluation frameworks that can be built into programmes from the beginning to ensure trackable impacts (http://www.fic.nih.gov/About/Staff/Policy-Planning-Evaluation/Pages/evaluation-framework.aspx). Long-term impacts of successful capacity building include cultural competency of staff and faculty; increased involvement of staff and faculty in global health brain research; the extent to which former trainees hold positions of influence in their countries; leadership of former trainees in research and research collaborations; and increased knowledge of disorders and their significance locally and internationally.

## CONCLUSIONS

**Figure 1:**
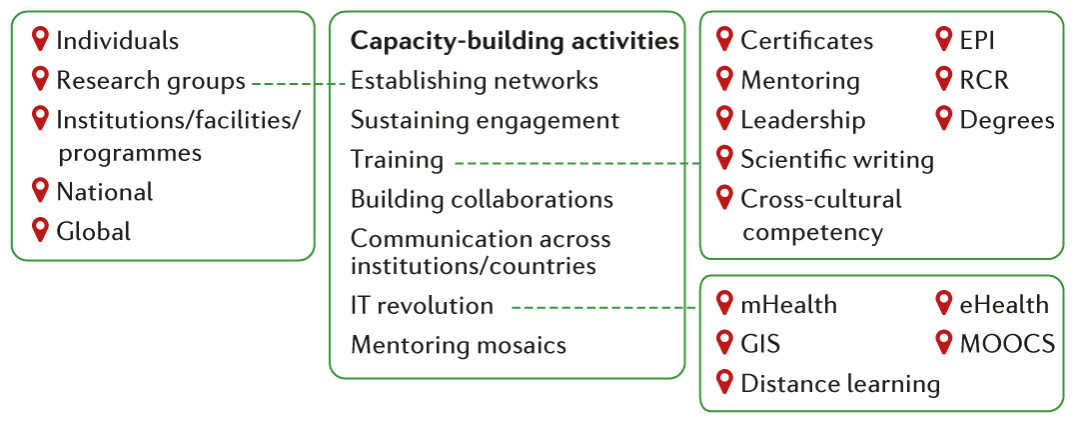
Research-capacity building activities can be achieved in a number of ways. Highlighted are examples of how three of activities can be achieved. EPI, epidemiology; GIS, geographical information systems; MOOCs; massive open online courses; RCR, responsible conduct of research.

Figure 1 summarizes some of the frameworks, components, pathways and tools involved in research-capacity building. As described, research-capacity building starts at the individual level. Although partnerships between high-income and LMICs are important, the goal is for research training, as well as research itself, to increasingly take place at the LMIC sites and for those sites to become research and training hubs in their own right.

Concurrent research capacity strengthening at the institutional and national levels is necessary to ensure research and career opportunities for, and the retention of, trained researchers. With respect to NMDS disorders in particular, targeted programmes provide opportunities for LMIC clinicians, faculty and trainees to gain new skills for conducting relevant research and to contribute to long-term sustainability of research conducted in LMICs (as the trainees become the trainers and attention is paid to institutional strengths and weaknesses). Although challenges exist, they can be managed and eventually reduced or overcome using principles and models learned and shared across programmes^8,33^. Robust evaluations of capacity-building activities with quantitative and qualitative measures should be conducted, shared and used to identify the most successful approaches and to al-low iterative improvements in individual, institutional and national level NMDS research capacity.

## Supplementary Material

Supp

## Figures and Tables

**Table 1:** Approaches to building research capacity

Category	Activity	General and specifc needs	**Anticipated benefts**	**Specifc approaches**
**Human capacity**	Increase the number of clinician researchers	Increased number of physicians and allied health professionals in research benefts all neuroscience researchers, including neurologists, neurosurgeons, infectious disease specialists, psychiatrists and other mental health practitioners As the incidence of neurological conditions increases, so will the need for more trained neurologists involved in research	Practical experience and opportunities for future training^12^	Create protected time and funding for research Decrease brain drain by investing in research and jobs in neurological areas Research methodology training during graduate and post-graduate medical training
	Increase research capacity of clinicians and researchers through workshops and short courses; and advanced degrees in public health (for example, epidemiology, biostatistics, clinical trials, health services and implementation science), clinical and basic science research Sub-specialized training on specifc skills related to nervous-system disorders	As the neurosciences have not received as much attention from research training grants, most areas would beneft from increased funding for research training Health-systems research is needed, which necessitates training in bioethics, research methodology, epidemiology, clinical trials, population-based methodology and intervention studies Specifc areas of neuroscience with unique needs, include mental health for which health-services research is crucial to increase the capacity of care services Many countries do not have the ability to diagnose neurogenetic conditions, and cannot provide genetic counselling or treatment	Address the shared burdens of common conditions, including neurodegenerative disorders, stroke and epilepsy Development of multidisciplinary teams of health-care professionals to improve prevention, pre-hospital care, and clinical care in neuroscience, such as trauma, mental health or neurogenetics	Multidisciplinary training and research The Wellcome trust-DBT India alliance fellowship for clinicians and research scientists Innovation in science pursuit for inspired research programme Initiative in neuroclinical research education The African Brain Mapping and Therapeutics Initiative, which advances neuroscience research in Africa by promoting global partnerships for brain-disease prevention and treatment
	Institutionalization of mentorship training	Outstanding mentoring is a prerequisite of any successful research-training programme Mentors must be expert in particular areas of research, such as cognitive assessment scales for the study of dementia-associated conditions, or seizure management for studies of epilepsy	Capacity for conducting neuroscience research will increase as trainees move into positions where they will start mentoring subsequent generations of trainees Increasing numbers of scientists and the development of research teams, research culture and an increase in scientifc literature and novel research	Wide spread mentorship training (for example, through programmes such as those of the NIH Fogarty International Center http://www.fc.nih.gov/)
**Infrastructure** **and tools**	Neuroimaging (for example, computerized tomography or magnetic resonance imaging)	Most neurological conditions require neuroimaging to confrm a diagnosis, disease stage or to monitor progress	Increased availability of neuroimaging will lead to better defnition of the burden of many neurological conditions, such as stroke, CNS infections, developmental, degenerative and genetic disorders, and trauma	Mentoring in the tools needed through fexible research and research-training programmes (for example through the Fogarty Global Brain programme)
	Genomic sequencing to detect SNPs in GWAS	GWAS are used to identify genetic variations (SNPs) associated with neurological and psychological disorders, including addiction	Detection of specifc genes through GWAS can lead to a better understanding of the functional mechanisms that are biologically important in disease pathogenesis and, ultimately, to better treatments for neurological diseases	The US National Center for Biotechnology Information has developed the Database of Genotype and Phenotype, where genetic sequencing information can be deposited and accessed
	Increased laboratory capacity	Most studies of neurological diseases require at least a basic laboratory to process blood, cerebrospinal fuid or other human samples With increasing complexity of studies, additional equipment is needed, such as polymerase chain reaction for detecting infectious pathogens or biosensors to detect environmental toxins	Many technologies introduced to increase laboratory capacity are also useful for diagnosing non-neurological diseases	Enhancement of laboratory capacity ofen requires the upgrade of electrical systems, and with larger laboratories may also require installation of air-conditioning systems
	Culturally appropriate assessment and screening tools	WHO and NIH databank of valid and reliable assessments for young people and adults. Each has an armamentarium of tools that are culturally appropriate Stigma, social and health disparities are more common with disorders such as epilepsy and schizophrenia	Disorders such as epilepsy and schizophrenia would beneft from increased recognition of barriers identifed through culturally appropriate screening tools	NIH and WHO promote scientifc discovery, and shared resources, that allow for data harmonization across many programmes Network meetings with special interest groups value the use of unifed concepts of addiction and mental health, from DSM to ICD classifcations.
**Technology**	Incorporation of emerging POC diagnostics from both the development of cross-cultural tools to the use of the tools	POC diagnostics could permit rapid diagnosis of many neurological infections in the feld, resulting in improved recognition and treatment POC diagnostics can be used to non-invasively monitor seizures, cerebral blood fow or intracranial pressure, but are not widely available for use in LMIC settings	Miniaturization of diagnostic technologies for genomics, infectious agents and environmental markers will enable a better understanding of gene–environment interactions and lead to new therapeutic approaches	Better sharing of data and instrumentation is needed, as well as collaborative grants
	Access to electronic scientifc literature	Common to all research is the need for understanding past and current scientifc literature	Improved access to electronic scientifc literature should lead to more scientifcally sound research and ofen leads to the creation of journal clubs, which in turn strengthens the culture of research	The HINARI Access to Research in Health programme provides free or low cost access to 200 neuroscience journals for not-for-profts in LMICs, but the top ranking 50 journals are not available Open access journals are available to everyone
	Introduction of mHealth technologies and e-learning strategies	Adoption of mobile technologies for surveillance, assessments and treatment are particularly needed in LMICs where cell phone ownership is rising rapidly, but access to conventional health care and health-care providers is limited Modular Internet-based curricula can be adapted for training for advancing neuroscience research Low-tech clinical simulation training emphasizing early life-saving interventions and procedures	Reaching patients with disabling neurological conditions using cell phones may prove easier than conventional methods for providing health information	Share resources Ofer classes for students at reduced cost
	Increase Internet capacity	Adapt information and communication technologies to support research and research-training programmes	Video conferencing for direct communication between mentors, colleagues and training in diverse settings	Communication technologies include Skype, GoToMeeting, AdobeConnect, WhatsApp, Polycom and WebEx
**Funding**	Pilot awards for LMIC researchers	Funding for research in LMICs is limited, but funding for neurological disease research is even more so	Providing funding to support pilot studies to LMIC colleagues and trainees should lead to increased research relevant to the LMIC setting and provide pilot data on which larger grant applications could be developed	Mentor LMIC partners through application processes.
	Increase governmental funding for research through universities and research institutions	Funds from national and international NGOs can increase research opportunities	Collaboration with foreign partners provides new research opportunities and support	PEPFAR, UNAIDS, WHO, and the Bill and Melinda Gates Foundation have made drugs and services signifcantly more accessible
	Research frameworks to support the implementation of the outcomes of well-designed studies relevant to neurological diseases	Evidence-based public health strategies to incorporate child neurodisability screening, clinical evaluation and rehabilitation packages into the health-care system	Maternal health programmes that work closely with early childhood programmes could ensure optimal pregnancy outcomes and develop efective interventions to enhance child development	
	Capacity building in translational science and knowledge management Learn to package the evidence in a format more accessible to policymakers in LMICs	LMIC partners are asking for translational science training	Infuence policymakers to redirect budget priorities to address brain disorders and their research Involve community partnerships	Involvement of the community, private sector and research sponsors during project planning establishes priorities, identifes research needs within the community, and identifes resources^34^

CNS, central nervous system; DSM, Diagnostic and Statistical Manual for Mental Disorder; GWAS, genome-wide association studies; ICD, International Classifcation of Diseases; LMIC, low- and middle-income countries; NGOs, non-governmental organizations; POC, point-of-care; SNP, single nucleotide polymorphisms; WHO, World Health Organization.
