# Supplementary material for: Building global capacity for brain and nervous system disorders research
Source: Nature. Author manuscript; Available in PMC 2017 Jan 12. (PMC5228466; doi:10.1038/nature16037)
Supplement: Supp [file NIHMS838953-supplement-Supp.pdf]

Table 1 | Selective programmes involved in long-term sustainable capacity building to promote health globally

Programme	Goals and strategies	Achievements
Canada Global Health Research–Capacity Strengthening Programs (GHR–CAPS) launched in 2010 in Asia, Africa, Latin America and the Caribbean http://www.ccghr.ca/	Builds a community of mentors in LMICs.<br>Trains doctoral and post-doctoral trainees.<br>Funds in-country research.	Doctoral students, post-doctoral fellows and summer scholars.<br>Interdisciplinary environment to benefit trainees and mentors.
Kenya Medical Research Institute (KEMRI), Wellcome Trust Research Programme (KWTRP) launched in 1989 in Africa http://www.kemri-wellcome.org/index.php/en	Provides post graduate training at master's, PhD and diploma level.<br>Provides post-doctoral support through mentorship and access to infrastructure.	Research findings integrated with Ministry of Health national health policy.<br>Disease burden in Kenya, Africa and other countries mapped.<br>Large scale health intervention studies that benefit the country.
Center for Global Mental Health (CGMH) launched in 2009 is a collaboration with the London School of Hygiene & Tropical Medicine in more than 30 countries worldwide http://www.centreforglobalmentalhealth.org/	Operates capacity building projects and consortia funded by NIH and US PEPFAR.<br>Offer master's and PhD courses in global mental health	More than 40 research projects in more than 30 LMICs.<br>Hub linking researchers and institutions globally to promote and influence mental health care services and research capacity
Consortium for Advanced Research Training in Africa (CARTA) launched in 2010 jointly-led by the Kenyan African Population and Health Research Center (APHRC) and South African University of the Witwatersrand in African countries http://www.cartafrica.org/	African led for partnerships inside and outside Africa: nine African universities, four African research institutes and seven northern academic institutions.<br>Builds local research capacity to understand determinants of health in Africa<br>Develops health infrastructure and interventions to promote health<br>Trains and retains a critical mass of African researchers<br>Promotes collaborative PhD programmes through scholarships<br>Provides post-doctoral training<br>Provides in-country advanced research grants	Research fellows enrolled in PhD programmes in five cohorts<br>External grant support<br>Network of over 600 researchers in Africa and other countries<br>200 in-country mentors trained
South Asian Hub for Advocacy, Research and Education on Mental Health (SHARE) launched in 2011 in South Asian countries http://www.sharementalhealth.org/	Builds mental health research capacity in South Asia through a collaborative program for International Research on Mental Health supported by the NIH.<br>Establishes a network of collaborating institutions to conduct research to inform local mental health policies.	Two administrative Hubs, one in Pakistan and one in India<br>Training opportunities for mental health researchers at partner institutions

NIH international focused programmes of individual institutes and centers http://www.fic.nih.gov/Global/Global-Health-NIH/Pages/default.aspx<br>NIH neurological, mental health and developmental disorders specific programmes at NIH http://www.fic.nih.gov/ResearchTopics/Pages/neurological-mental-disorders-diseases.aspx	Strengthen research capacity in brain and neurological health and disorders in LMICs by promoting LMIC focused research and research collaborations between individuals and institutions in high-income and LMICs and within LMICs.<br><br>Participate in trans-NIH programmes for research capacity building/training for LMICs.	Fogarty and the NIH invest in research to address the burden of neurological, mental health and developmental disorders to LMICs. For example: addiction; neurodegenerative diseases, such as Alzheimer's and Parkinson's; neurodevelopmental disorders, such as autism, fetal alcohol syndrome (FAS) and learning disabilities; neuropsychiatric conditions such as depression, schizophrenia, and post-traumatic stress disorder (PTSD); and seizure disorders.<br><br>Fogarty and NIH also support research on how environmental exposures, childhood adversity, nutrition, injury and other factors impact brain development, and how the rehabilitation process for these diseases and disorders influences treatment and recovery.<br><br>Research at NIH on the full spectrum of brain disorders and diseases spans many of the 27 institutes and centres.
**Fogarty International Center**<br><br>**Region:** All LMICs<br>www.fic.nih.gov	Coordinate a portfolio of:<br><br>Institutional research training programs focusing on masters, PhD and postdoctoral level training for LMIC investigators http://www.fic.nih.gov/Programs/Pages/chronic-lifespan.aspx<br><br>Research Programs focused on Global Health and NMDs http://www.fic.nih.gov/ResearchTopics/Pages/neurological-mental-disorders-diseases.aspx	Worldwide LMIC networks of NMD-related research training programs and growing LMIC cadre of NMD researchers.
National Institute of Mental Health (NIMH) international programmes around the world http://www.nimh.nih.gov/about/organization/gmh/office-for-research-on-disparities-and-global-mental-health-ordgmh.shtml	The NIMH Global Mental Health Research Program within the Office for Research on Disparities and Global Mental Health has emphasis in the following areas: equity in access to, quality of, and outcomes of mental health care worldwide; integration of mental health care into global health-care platforms in low-resource settings (for example, primary care, school health, chronic disease management, HIV/AIDs care, social services, and so on); research capacity building in low-resource settings (for example, researcher training and mentoring, development of research infrastructure) to develop a multidisciplinary	LMIC research hubs<br><br>Worldwide network of mental health research training programs and research projects.

	mental health research workforce worldwide and to build sustainable regional bases for further research, networking, and evidence-based mental health policy.	
National Institute of Child Health and Human Development (NICHD) international programmes in the United States and LMICs.	Biomedical/Biobehavioural (LMIC) Research Administrator Development (BRAD) programme. LMIC Research Administration: The Bridge to Sustainable Research http://www.nichd.nih.gov/about/org/od/ohe/brad/Pages/overview.aspx	NA
National Institute on Drug Abuse (NIDA) international programmes http://www.drugabuse.gov/international<br><br>with a LMIC focus	NIDA international programme fellowships http://www.drugabuse.gov/international/fellowships-postdoctoral-training<br>Build partnerships with countries, organizations and individual researchers to promote new research initiatives, build international research capacity, and disseminate knowledge.<br>NIDA supports the international effort because the institute recognizes that addiction has no borders, and that no country can solve the problem by acting alone.	Worldwide network of international investigators http://www.drugabuse.gov/international/fellows-world-map
NIH coordinated Medical Education Partnership Initiative (MEPI)<br><br>launched 2010 in sub-Saharan Africa http://www.pepfar.gov/partnerships/initiatives/mepi/index.htm	Builds clinical and research capacity by strengthening medical education institutions through PEPFAR support to retain researchers and clinicians in country.<br>Develops regional training centres.<br>Facilitates distance learning and resource sharing between institutions.	Fundamental changes in the approaches to medical education<br>Changes in curricula, content and teaching format.<br>Use of technology to network with other medical schools locally and internationally.<br>Twelve African countries with a network of more than 30 regional partners and over 20 foreign collaborations.
Australian AusAID http://dfat.gov.au/people-to-people/australia-awards/Pages/australia-awards-scholarships.aspx http://cimh.unimelb.edu.au/research_and_publications/imhr/ausaid_linkages_program	Builds research infrastructure by providing scholarships to students from LMICs.<br>Supports AusAID public sector linkages programme in Indonesia.	
Grand Challenges Canada http://www.grandchallenges.ca/grand-	Saving Brains supports bold ideas to improve early brain and	Total investment of Saving Brains to date is US$41 million invested in 107 projects and a platform to

challenges/<br>Current partners are Canadian Government, Aga Khan Foundation, Bernard van Leer Foundation, Bill & Melinda Gates Foundation, Maria Cecilia Souto Vidigal Foundation, Norlien Foundation, UBS Optimus Foundation and World Vision Canada	child development in LMICs<br>http://www.grandchallenges.ca/saving-brains/	accelerate progress against the challenges.<br>Total investment of Global Mental Health to date is $32 million in 64 projects throughout Africa, Asia, and South and Latin America/the Caribbean and the Mental Health Innovation Network.<br>http://www.grandchallenges.ca/grand-challenges/global-mental-health/
UNICEF, World Bank, World Health Organization, United Nations Development Programme in specific LMICs	Promotes research capacity building in LMICs in health and mental health through various programmes.	NA
UK Wellcome Trust in specific LMICs<br>http://www.wellcome.ac.uk/Funding/index.htm	Advances research infrastructure and builds capacity in health and mental health in LMICs.	Has played an important role in many LMICs to give independence to trained investigators who can implement their own studies
